# Delayed discharge from intellectual disability in-patient units

**DOI:** 10.1192/pb.bp.113.044388

**Published:** 2014-10

**Authors:** John Devapriam, Satheesh Gangadharan, Judith Pither, Matthew Critchfield

**Affiliations:** 1 Leicestershire Partnership NHS Trust

## Abstract

**Aims and method** We undertook a cross-sectional service evaluation of the reasons and extent of delay in the discharge process in an intellectual disability hospital over a 12-month period. Delays at each stage of the discharge process are also quantified in this study.

**Results** We found that discharge was delayed for 29% of patients during the study period. The majority (78.5%) was due to awaiting completion of assessment of future care needs and waiting for public funding.

**Clinical implications** Commissioners (health and social), provider trusts, regulators and community providers should consider the reasons for delay in the discharge process and adopt a whole systems approach to discharge planning. This is highly relevant in light of recommendations by the Department of Health following the Winterbourne View scandal, which has raised concern about patients staying in intellectual disability in-patient units too long and for the wrong reasons.

Delayed discharge (also called delayed transfer of care or bed blocking) refers to a situation where a patient is deemed medically fit for discharge but they are unable to leave hospital because arrangements for continuing care have not been finalised.^1^ It was a concern particularly for older people in acute hospitals at the turn of the century, which prompted the Health Select Committee of the House of Commons to examine the problem in detail.^2^ The Committee report describes delayed discharges as a symptom of poor bed management in hospitals and a failure in the interface working between health and social care.

The Department of Health published its good practice guidance on delayed discharges in 2003^3^ and this was followed by the Community Care (Delayed Discharges etc.) Act 2003.

However, this legislation did not extend to mental health or intellectual disability services (also known as learning disability services in the UK) and therefore delayed discharges in these services are not monitored or scrutinised, as they are in acute hospitals. Several studies^4-7^ and national inquiries^8-10^ have highlighted the problem of length of stay and delayed discharges of patients in intellectual disability in-patient units.

In light of the recent changes in commissioning frameworks in the National Health Service (NHS) and the concerns over the impact of social care funding cuts on the NHS,^11^ this study was undertaken to identify the factors associated with delay in discharge from a specialist intellectual disability in-patient unit.

## Method

The study was conducted in a specialist in-patient unit for people with intellectual disabilities. The unit has 16 beds, 8 of which are acute admission beds (category 2 beds)^12^ and 8 are for rehabilitation purposes (category 4 and 5 beds).^13^ The unit receives input from a multidisciplinary health team, which includes nurses, speech therapists, occupational therapists, a psychologist and a psychiatrist. It is part of a wider intellectual disability service, which provides community services, short breaks and autism day care for people with intellectual disability.

**Fig 1:**
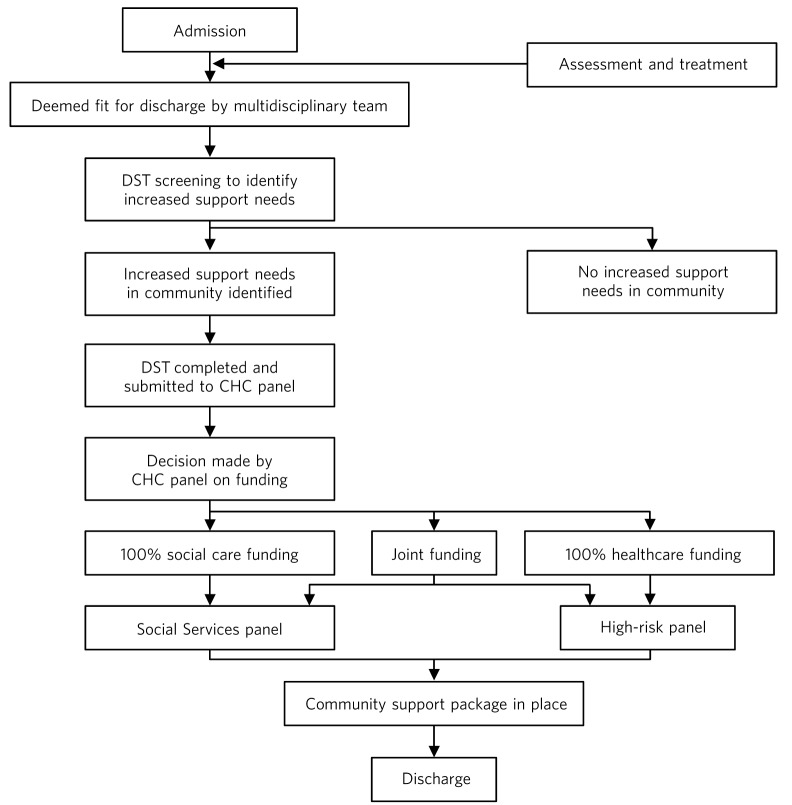
Discharge process in intellectual disability in-patient hospitals. CHC, continuing healthcare; DST, Decision Support Tool.

The in-patient team implemented a care pathway-based approach to assessment and treatment within the unit. The approach adopted an evidence-based, multi-agency and lean method to reduce waiting times, reduce length of stay and improve the quality of care within the unit. This approach increased the productivity within the unit and the turnover of patients, with reduction in length of stay, and produced positive outcomes for patients. However, there remained a group of individuals for whom the length of stay in hospital after being certified as fit for discharge has been long. Anecdotal evidence suggested that this delay in discharge is due neither to patient characteristics nor to a unit’s processes in the assessment and treatment, but is due to system issues in the discharge process. The process followed once a patient is deemed fit for discharge is shown in Fig. 1.

Data were obtained from the delayed discharge database held by the service. All patients who have been an in-patient at any point between 1 February 2012 and 31 January 2013 were identified. Out of this sample, patients who have been coded as delayed discharge were identified and more information was collected using a template provided by the Department of Health (SITREPS).^14^ Although patients may have several reasons for the delay, according to the guidelines,^14^ only one (most relevant) reason was taken into account. Further analysis was undertaken to establish how long (in bed days) the patients spent in hospital after being deemed fit for discharge and how many bed days in total were spent at each stage of the discharge process (as illustrated in Fig. 1). Patient details were otherwise anonymous. The study was a service evaluation and therefore did not require ethics committee approval.

## Results

The total number of patients who received care and treatment in the 16-bed in-patient unit from 1 February 2012 to 31 January 2013 was 49. Of these, 14 (29%) were recorded to have been delayed discharges (Table 1).

When analysing reasons for delay, the results show that discharge of 7 patients (50%) was delayed because they were still awaiting completion of an assessment of future care needs and identification of an appropriate care setting. The number of bed days spent as delayed discharge in this category ranges from 72 to more than 365 days (which means that some of these patients were still not discharged when this study was undertaken). Out of 14 patients, 4 (28.5%) were delayed because they were awaiting funding agreement either from Social Services or NHS continuing healthcare, or both, with bed days as delayed discharge ranging from 50 to more than 424. One patient (7.1%) was delayed for more than 270 days because there was no appropriate placement available for them in the community. For one patient (7.1%), their family exercised their right to choose an appropriate placement in the community, leading to a delay of 72 days. In one other patient (7.1%), the delay was due to ‘other reasons’, which included legal proceedings taken by the local authority to place an individual in the community.

The average number of bed days spent by the 14 patients at each stage of the discharge process (as outlined in Fig. 1) is given in Table 2. The results show a breakdown of the delay involved at each stage when it is identified that the patient requires an increased support package in the community.

**Table 1:** Reasons for delay (total sample size *n* = 14)

Reason for delay	Delays, *n* (%)	Bed days in delayed discharge, *n* (range)
Awaiting completion of an assessment of future care needs and identifying an appropriate care setting	7 (50)	72 to 365+
		
Awaiting Social Services funding for residential or home care; includes cases where Social Services and NHS have failed to agree funding for a joint package, or an individual is disputing a decision over fully funded NHS continuing care in the independent sector	4 (28.5)	50 to 424+
		
Awaiting further NHS care	0 (0)	0
		
Awaiting care home placement	1 (7.1)	270+
		
Awaiting domiciliary package (including home adaptations and equipment)	0 (0)	0
		
Patient and/or their family exercising their right to choose a residential or nursing home under the Direction on Choice, following the agreement of Social Services funding Where patients who will be funding their own care are creating an unreasonable delay in finding a place (e.g. through insisting on placement in a home with no foreseeable vacancies)	1 (7.1)	72
		
Other reasons (including housing)	1 (7.1)	945+

NHS, National Health Service.

**Table 2:** Delay in stages of discharge process (*n* = 14)

Stage	Description	Bed days Average *n* (range)
Stage 1	From the date when the patient is deemed fit for discharge until the date when the DST is submitted to CHC panel	121 (0-910)
		
Stage 2	From the date the DST is submitted to when the CHC panel makes a decision on proportion of funding from CHC	65 (8-300)
		
Stage 3	From the date a funding stream decision is made by CHC to the date when respective health and/or local authority panels authorise the funding	68 (0-455)
		
Stage 4	From the date funding is authorised to the date of actual discharge from hospital	29 (6-90)

CHC, continuing healthcare; DST, Decision Support Tool (a national tool used to identify what proportion of a patient’s funding package is to be funded by NHS continuing healthcare).^13^

## Discussion

### Main findings

The study showed that 29% of patients in the sample were deemed to be delayed discharge. This replicates previous findings in intellectual disability hospitals (17.5% ^6^ to 55%^5^), but is higher than the delayed discharge rates in acute hospitals (6%).^2^ The majority of patients were delayed due to delay in completion of future care needs, delay in identifying an appropriate community placement and delay in funding. Surprisingly, only one patient was delayed due to lack of availability of an appropriate placement in the community; the rest had existing community placement identified and only one other patient had to wait for a bespoke placement to be commissioned. This reiterates that the reason for delay in most cases is a system issue rather than a lack of available placements for complex care in the community.

The extent of delays in each stage of the discharge process raises some discussion points.

- The delay in stage 1 is due to Decision Support Tool (DST)^13^ not submitted in a timely manner. This has been caused by changes in commissioning NHS continuing healthcare where DSTs were requested to be redone for some individuals who were deemed to be entitled to 100%-funded healthcare. Although it is good practice to start planning discharge once a patient is admitted, this is practically not possible as the lead commissioner (who oversees placing an individual in the community - health or social care) cannot be identified unless a funding decision is made. This funding decision cannot be made unless a DST is completed, which cannot be completed until a patient is stable (medically fit).
- The delays in stage 2 are to do with delays in triaging and making a decision on funding proportion from continuing healthcare. Department of Health guidance^15^ states that in most cases DSTs should be heard at panel within 28 days. However, our findings suggest that this is not the case. Another issue is that DSTs could become out of date while waiting for the panel to consider them.
- The delays in stage 3 are to do with the delay in health and local authority panels (high-risk and quality assurance panels respectively) to authorise the payments.
- The delays in stage 4 result from identification of a lead commissioner (following the funding decision in stage 3), identifying a placement and support providers. There can also be a delay due to community providers recruiting staff, training them and making adaptations to the accommodation prior to the patient’s discharge.

### National scenario and findings from other studies

Delayed discharge is a major problem in mental health and intellectual disability services. The Department of Health guidance on delayed transfer of care^3^ and the Community Care (Delayed Discharges etc.) Act 2003 placed new duties on local authorities and the NHS to work together, encourage clear communication, improve assessment and provision of community care for patients discharged from acute hospitals, encourage the development of new services to enable this, prevent unnecessary admissions and promote patient independence.^16^ However, these duties do not extend to mental health or intellectual disability services. This, we feel, is one of the reasons why this area has not been monitored or scrutinised as it is in acute hospitals. Following the Winterbourne abuse scandal,^17^ it has been found that nationally there are a lot of people with intellectual disability and mental health or behaviour problems who are in such in-patient units inappropriately and for long periods of time.^10^ One of the recommendations by the Department of Health^10^ is to set up a joint review team led by local authority and health commissioners to review individuals who are staying for long periods of time in in-patient units and to have a plan of action to move them into community settings. However, we feel that this will continue to be a problem unless discharge processes are aligned between health and social care systems and appropriate placements are commissioned in the community.

Several studies have highlighted the problem of delayed discharge of patients from these units and have identified several associated factors. These include certain patient characteristics such as being ‘less able’, older, ‘more challenging’,^4^ diagnosed with dementia or psychosis, having family history of psychiatric illness and repeated admissions in the past,^5^ availability of community provision of services,^6^ variables related to the discharge process, which includes health and social care commissioning,^6^ and poor inter-agency communication.^7^ Between 17.5 and 55% of patients are delayed discharges in these units.^5,6^ The Healthcare Commission report, *A Life Like No Other*,^8^ states that nearly 20% of patients in hospitals were receiving no active treatment and that there was a lack of plans for them to be discharged.

The reasons for delay associated with commissioning processes have previously been classified into three different categories.^18^ First is the ‘devolved approach’, where health authorities devolve responsibility for resettlement after acute admission to local providers such as the local resettlement teams, community intellectual disability teams or Social Services departments. Second is ‘no approach at all’, either because the authority was in the midst of a reorganisation or because they had no effective mechanism to review patients for resettlement. The third approach was ‘the clinical approach’, where the authorities employed a resettlement officer who reviewed the needs of patients on a case-by-case basis and worked proactively with the NHS trust, local authorities and local providers to identify placements for these patients. It was found, unsurprisingly, that health authorities who adopted the last approach had been the most effective in reducing delayed discharges. Cumella *et al* make two useful recommendations on effective inter-agency working to prevent delayed discharges. The first is ‘improved bed management’, which includes preventive work to avoid inappropriate admissions to an in-patient facility and to introduce systematic and proactive discharge planning once admitted. They also suggest intermediate placements jointly funded by health and social care services for patients to move on from in-patient units which will not be as resource intensive as an in-patient unit. The second recommendation they make is to develop a wider range of community-based accommodation and the need for a whole systems approach with joint investment by health and Social Services authorities.

The true cost of delayed discharges in monetary terms to the NHS is not known. In 2002, the Health Select Committee estimated the average cost of an acute hospital bed to be £120 000 per year,^2^ and assuming that there are approximately 6000 beds occupied by patients who should have been discharged, this equates to an annual cost of £720 million. In mental health and intellectual disability services, it is even more difficult to establish the cost of in-patient beds and generalise it to different services, due to the variability of service provision and commissioning methods.

### Implications for future practice and policy

#### Implications for in-patient providers

- A care pathway-based approach to assessment and treatment involving community teams and commissioners should be practised. This will ensure that appropriate admission will take place with discharge planning right from the stage of admission. This will also ensure that interventions are delivered timely.
- There should be joint training for health and social care staff on completing the DST to ensure that the relevant information is submitted with multidisciplinary agreement.
- The role of advocates for patients within in-patient units should include challenging the delay in discharge process in the best interests of patients.
- Patients and their carers should be involved in discharge planning.

#### Implications for local authorities

- Allocation of a social worker specifically for the in-patient unit will ensure that all social issues and needs are assessed right from admission.
- There should be streamlining of funding decisions within social care.
- Social workers who are trained in intellectual disability should be allocated to be case managers.
- There is a need for market research and development of appropriate placements and providers, with monitoring of the funding needs.

#### Implications for clinical commissioning groups

- Streamlining of the continuing healthcare process and decision-making is required.
- To aim to provide decisions on DSTs within the target of 28 days set by the Department of Health.
- Joint development of appropriate community placements with local authorities.
- A key professional should be appointed jointly by health commissioners, local authorities and provider trusts to take on the role of pathway coordination to facilitate discharge for patients.

### Limitations of the study

The study is limited to local service structure and funding streams, although it reflects the national picture. The sample size is small and therefore the study relies on a descriptive methodology. We recommend that more policy research is required, involving multiple centres with different models of in-patient services and community service provision, on the effects that delayed discharge has on patients’ mental and physical health and the direct and indirect costs associated with this issue.

## References

[1] Bryan K Gage H Gilbert K Delayed transfers of older people from hospital: causes and policy implications. Health Policy 2006; 76: 194–201 16040152 10.1016/j.healthpol.2005.06.005

[2] House of Commons Health Select Committee. Delayed Discharges (Third Report of Session 2001-02). TSO (The Stationery Office), 2002

[3] Department of Health. Discharge from Hospital: Pathway, Process and Practice. Department of Health, 2003

[4] Watts RV Richold P Berney TP Delay in the discharge of psychiatric in-patients with learning disabilities. Psychiatr Bull 2000; 24: 179–81

[5] Dickinson MJ Singh I Mental handicap and the new long stay. Psychiatr Bull 1991; 15: 334–5

[6] Perera C Simpson N Douds F Campbell M A survey of learning disability in-patient services in Scotland in 2007. J Intellect Disabil 2009; 13: 161–71 19628535 10.1177/1744629509339091

[7] Patterson T Higgins M Dyck DJ A collaborative approach to reduce hospitalisation of developmentally disabled clients with mental illness. Psychiatr Serv 1995; 46: 243–7 7796210 10.1176/ps.46.3.243

[8] Healthcare Commission. A Life Like No Other: A National Audit of Specialist Inpatient Healthcare Services for People with Learning Difficulties in England. Healthcare Commission, 2007

[9] Department of Health. Department of Health Review: Winterbourne View Hospital (Interim Report). Department of Health, 2012

[10] Department of Health. Transforming Care: A National Response to Winterbourne View Hospital. Department of Health Review: Final Report. Department of Health, 2012

[11] NHS Confederation. Papering Over the Cracks: The Impact of Social Care Funding on the NHS. NHS Confederation, 2012

[12] Royal College of Psychiatrists’ Faculty of Psychiatry of Intellectual Disability. *People with Learning Disability and Mental Health, Behavioural or Forensic Problems: The Role of In-Patient Services* (Faculty Report FR/ID/03). Royal College of Psychiatrists, 2013

[13] Department of Health. Decision Support Tool for NHS Continuing Healthcare (Revised). Department of Health, 2012

[14] Department of Health. SITREPS 2003-2004 Final Version Definitions and Guidance. Department of Health, 2003

[15] Department of Health. National Framework for NHS Continuing Healthcare and NHS Funded Nursing Care. Department of Health, 2012

[16] Personal Social Services Research Unit. *Delayed Discharges from Hospital* (Discussion Paper M197). PSSRU, 2007

[17] BBC One Panorama. Undercover Care: The Abuse Exposed. BBC, 2011 Available at http://www.bbc.co.uk/programmes/b011pwt6

[18] Cumella S Marston G Roy A Bed blockage in an acute admission service for people with a learning disability. Br J Learn Disabil 1998; 26: 118–21

